# Supplementary material for: Carbohydrate, Lipid, and Apolipoprotein Biomarkers in Blood and Risk of Thyroid Cancer: Findings from the AMORIS Cohort
Source: Cancers (Basel). 2023 Jan 14;15(2):520. doi: 10.3390/cancers15020520 (PMC9856513; doi:10.3390/cancers15020520)
Supplement: Supplementary file 1 [file cancers-15-00520-s001.zip › supplementary table S1.pdf]

cancers MDPI

Table S1. Incidence rates (IR) per 100,000 person-years and hazard ratio (HR) with 95% confidence interval (CI) of thyroid cancer per SD increase of blood biomarkers of lipid, carbohydrate, and apolipoprotein metabolism, analysis restricted to first blood samplings in relation to an occupational health check-up

Biomarker	N of cases	IR	HR (95% CI)[a]
Glucose	325	5.0	1.07 (0.95-1.20)
TC	349	5.1	0.93 (0.82-1.05)
LDL-C	140	5.1	0.99 (0.82-1.19)
HDL-C	138	5.1	0.82 (0.67-0.99)
LDL-C/HDL-C[b]	138	5.1	1.07 (0.96-1.18)
TG[b]	349	5.1	1.07 (0.96-1.21)
ApoA-I	113	4.9	0.86 (0.69-1.07)
ApoB	98	4.7	1.10 (0.89-1.35)
ApoB/ApoA-I[b]	98	5.0	1.13 (0.90-1.38)

[a] Analyses were adjusted for sex, age at first blood sampling, fasting status at first blood sampling, occupational status, and country of birth.

[b] Logarithmic transformation (log2) was used to the variables of TG, LDL-C/HDL-C ratio, and ApoB/ApoA-I ratio.

Abbreviations: TC, total cholesterol; LDL-C, low-density lipoprotein cholesterol; HDL-C, high-density lipoprotein cholesterol; TG, triglycerides; ApoA-I, apolipoprotein A-I; ApoB, apolipoprotein B.
