# Supplementary material for: Carbohydrate, Lipid, and Apolipoprotein Biomarkers in Blood and Risk of Thyroid Cancer: Findings from the AMORIS Cohort
Source: Cancers (Basel). 2023 Jan 14;15(2):520. doi: 10.3390/cancers15020520 (PMC9856513; doi:10.3390/cancers15020520)
Supplement: Supplementary file 1 [file cancers-15-00520-s001.zip › supplementary table S2.pdf]

cancers MDPI

Table S2 Incidence rates (IR) per 100,000 person-years and hazard ratio (HR) with 95% confidence interval (CI) of thyroid cancer per SD increase of blood biomarkers of lipid, carbohydrate, and apolipoprotein metabolism, analysis restricted to first blood samplings after overnight fasting.

Biomarker	N of cases	IR	HR (95% CI)[a]
Glucose	325	5.1	1.11 (0.99-1.24)
TC	340	5.2	0.95 (0.84-1.08)
LDL-C	140	5.2	0.93 (0.77-1.12)
HDL-C	139	5.2	0.83 (0.69-1.00)
LDL-C/HDL-C[b]	139	5.2	1.03 (0.87-1.22)
TG[b]	341	5.2	1.04 (0.92-1.18)
ApoA-I	122	5.0	0.89 (0.73-1.08)
ApoB	111	4.8	1.01 (0.83-1.24)
ApoB/ApoA-I[b]	111	5.0	1.07 (0.87-1.31)

[a] Analyses were adjusted for sex, age at first blood sampling, fasting status at first blood sampling, occupational status, and country of birth.

[b] Logarithmic transformation (log2) was used to the variables of TG, LDL-C/HDL-C ratio, and ApoB/ApoA-I ratio.

Abbreviations: TC, total cholesterol; LDL-C, low-density lipoprotein cholesterol; HDL-C, high-density lipoprotein cholesterol; TG, triglycerides; ApoA-I, apolipoprotein A-I; ApoB, apolipoprotein B.
